# The MDAR (Materials Design Analysis Reporting) Framework for transparent reporting in the life sciences

**DOI:** 10.1073/pnas.2103238118

**Published:** 2021-04-23

**Authors:** Malcolm Macleod, Andrew M. Collings, Chris Graf, Veronique Kiermer, David Mellor, Sowmya Swaminathan, Deborah Sweet, Valda Vinson

**Affiliations:** ^a^Edinburgh CAMARADES group, Centre for Clinical Brain Sciences, The University of Edinburgh, Edinburgh EH8 9YL, United Kingdom;; ^b^eLife Sciences Publications Ltd., Cambridge CB4 1YG, United Kingdom;; ^c^John Wiley & Sons, Oxford OX4 2DQ, United Kingdom;; ^d^Public Library of Science, San Francisco, CA 94111;; ^e^Center for Open Science, Charlottesville, VA 22903;; ^f^Nature Portfolio, Springer Nature, New York, NY 10004;; ^g^Cell Press, Cambridge, MA 02139;; ^h^Science, American Association for the Advancement of Science, Washington, DC 20005

Transparency in reporting benefits scientific communication on many levels. While specific needs and expectations vary across fields, the effective interpretation and use of research findings relies on the availability of core information about research materials, study design, data, and experimental and analytical methods. For preclinical research, transparency in reporting is a key focus in response to concerns of replication failure. The inconsistent reporting of key elements of experimental and analytical design, alongside ambiguous description of reagents and lack of access to underlying data and code, has been shown to impair replication (1) and raise doubt about the robustness of results (2, 3). In response to early concerns about replication of published results, funders, publishers, and other stakeholders have called for improvements in reporting transparency (4–7). Several initiatives ensued, including journal policies and joint efforts by journals, funders, and other stakeholders (8–10). One of these initiatives, the Transparency and Openness Promotion (TOP) guidelines (11), outlines a policy framework at the journal level that over 1,000 journals and publishers have adopted.

The National Academies have focused on reproducibility and replicability* challenges through several recent initiatives leading to consensus reports, including *Reproducibility and Replicability in Science* (12), *Open Science by Design: Realizing a Vision for 21st Century Research* (13), and *Fostering Integrity in Research* (14). Each of these reports concludes that lack of reporting transparency is one factor which contributes to these systemic problems. Building on these findings, the National Academies convened a public workshop in September 2019 titled “Enhancing Scientific Reproducibility in Biomedical Research Through Transparent Reporting.” The workshop was designed to discuss the current state of transparency in reporting biomedical research and to explore the possibility of improving the harmonization of guidelines across journals and funding agencies.

During this workshop, we provided a preliminary description of our MDAR initiative, the Materials Design Analysis Reporting Framework (15). Our MDAR working group came together in late 2017 with the intention to develop a policy framework to promote transparent reporting that would support article-level application of the principles of the TOP guidelines, with a focus on implementation through journal policies and editorial practice. Our goal was to develop a framework and an implementation support system that would be practically applicable for a broad range of journals in the life sciences. In many ways, the MDAR Framework is a joint iteration on our collective previous experience with guidelines, checklists, and editorial requirements, toward a harmonized and practical guideline for minimum reporting requirements.

The National Academies of Science, Engineering, and Medicine (NASEM) workshop provided a valuable opportunity for broader consultation across relevant stakeholder groups (16). We incorporated the feedback we received at the workshop, and here we report the results of our project in the form of three main MDAR outputs: the MDAR Framework, which forms the core policy recommendation, and the Checklist and Elaboration documents, which support its implementation (Box 1). We present these outputs as a contribution to help life sciences journals achieve the opportunities highlighted during the NASEM workshop through a pragmatic and harmonized approach.

**Box 1:** Description of MDAR outputs

MDAR output	Description of MDAR output
MDAR Framework	The Framework sets out minimum requirements and best-practice recommendations across four core areas of Materials (including data and code), Design, Analysis, Reporting (MDAR). https://osf.io/xfpn4/
MDAR Checklist	The checklist is intended as an optional tool to help operationalize the Framework by serving as an implementation tool to aid authors complying with journal policies and editors and reviewers in assessing reporting and adherence to the standard. https://osf.io/bj3mu/
MDAR Elaboration document	The Elaboration document is a user guide providing context for the MDAR Framework and Checklist and recommendations on how to use them and tracking the MDAR development process and rationale. https://osf.io/xzy4s/

## Devising a Harmonized Reporting Framework

In devising this Framework, we drew not only on the collective experience of journal initiatives, including existing checklists, but also on the results of recent meta-research studying the efficacy of such interventions (17–19), existing transparency and reporting frameworks (20, 21), and the above-mentioned NASEM consensus reports (see *SI Appendix—MDAR Elaboration* for a complete list of baseline reference material). We had a strong focus on straightforward and pragmatic approaches to implementation and on balancing the demands of research users for improved reporting with reasonable expectations for authors, reviewers, and editors. Therefore, we have not attempted to make the Framework comprehensive, but we have consulted broadly to include the elements that, considering current practice, could bring the most notable improvements across a large number of journals.

The scope of the MDAR Framework was the life sciences, and within this our project had a strong focus on information that is relevant for exploratory laboratory research; MDAR is therefore likely to be more useful for some disciplines than for others. We also recognize the added value of many community-endorsed specialized guidelines that go further in their more focused expectations of more specific research areas. The MDAR Framework is not comprehensive and should not supersede any existing reporting standards. It is a generalist framework intended to be broadly applicable to research in the life sciences and to lift minimum expectations in a concerted way. MDAR is meant to be not only compatible with but also to be enhanced by existing specialist standards and guidelines (e.g., EQUATOR Network, FAIRsharing, and ARRIVE).

The organizing principles of the MDAR Framework centered around three types of information: accessibility, identification, and characterization. Accessibility refers to the disclosure of whether and how a specific Framework element (e.g., material, data, code, or protocols) is accessible, including any restrictions to that access. Identification refers to the information necessary for unique and unambiguous designation and provenance of each element (typically this includes a unique persistent identifier). Characterization refers to the minimum description of the item which is relevant for interpretation and replication.

For each element of the Framework, we have identified a basic minimum requirement as well as an aspirational set of best practices that provide directional guidance for the future evolution of the requirements. With time we expect that elements currently identified as “best practice” will instead be identified as a “basic minimum requirement” as reporting performance improves.

## A Road Test and Broad Consultation

New approaches to improving reproducibility and replicability bring with them costs of adoption and implementation. If the costs to journals outweigh the benefits they will stop using the MDAR Framework, but it is more difficult to account for the additional burden placed on authors. Authors do recognize that checklists can improve reporting quality (22), and this is supported, to an extent, by empirical research (17–19); a strategy of using simple checklists which incur the lowest burden is reasonable. Ultimately, improved reproducibility and replicability benefits future researchers as they attempt to build on previous work, so there is also broader value from such endeavors.

Previous experience highlighted the benefits not only of broad stakeholder consultation but also of testing interventions in “real-life” situations before implementation. We therefore reached out to the publishing community to recruit volunteer journals for a pilot study. Thirteen journals and publishing platforms participated: *BMC Microbiology* and *Ecology and Evolution*, *eLife*, EMBO journals, *Epigenetics*, F1000R, *Molecular Cancer Therapeutics*, *MicrobiologyOpen*, *PeerJ*, *PLOS Biology*, PNAS, *Science*, and *Scientific Reports*. As a proxy for testing the MDAR Framework, we tested its main implementation tool, the MDAR Checklist, a series of questions designed to determine whether a study adheres to the minimum requirements in the MDAR Framework. The participants were also given the MDAR Elaboration Document as a further guide to clarify expectations.

The pilot sought, first, to understand whether the MDAR Checklist described each reporting standard with sufficient clarity to guide the assessment of submitted manuscripts and, second, whether the Checklist was useful to authors seeking to meet journal policy expectations and to editors assessing manuscripts against those requirements.

Participating journal teams screened 289 manuscripts using the checklist; 89 of these were assessed twice, by independent journal assessors blinded to each other’s assessment, to determine interassessor agreement. We interpreted poor (no better than expected by chance) interobserver agreement as an indication of insufficient clarity and revisited the description of affected items in the Checklist and Elaboration document to clarify them. Specifically, there had been poor agreement around the provenance of cell lines, primary cultures, and microbes, in details of ethical approvals, and on whether criteria for exclusion had been determined prior to data collection. We suggest that implementing journals may wish to give particular attention to these items in the training of editorial staff. Only 15 of 42 items were considered relevant to more than half of the 289 manuscripts considered. To address this, a common suggestion from study participants was to organize the checklist in a nested way to allow users to easily skip over sections that are not relevant. While this is an excellent suggestion, we concluded that the best organization may be journal-specific and left this as an implementation decision for the journals electing to use a checklist.

Across all participating journals we sought feedback from a corresponding author of these 289 manuscripts (some manuscripts had more than one corresponding author), and from editorial staff members handing those manuscripts. Eighty percent of 211 corresponding authors who responded, and 84% of 33 editorial staff, found the checklist “very” or “somewhat” helpful.

We also obtained further feedback on the MDAR Framework, Elaboration document, and Checklist from 42 experts with experience in various aspects of reproducibility and replicability, including using and developing checklists to improve reporting standards. While incorporating this feedback and refining the Framework and associated outputs further we added and strengthened some requirements but we were careful to ensure that the minimum requirements remained reasonable and pragmatic to avoid undue burden on authors, given current practice.

## Endorsing and Adopting the MDAR Framework

We developed the MDAR Framework as an adaptable system allowing flexible adoption to meet the different needs of specific disciplines, community norms, and resource constraints. We envision that by endorsing the MDAR Framework journal signatories will express their support for transparent reporting principles outlined by the MDAR Framework and commit to taking steps to incorporate the MDAR guidelines into their journal practices.

Having endorsed the MDAR Framework, journals can choose from three increasingly stringent levels of implementation to give signatories flexibility, allowing a lower threshold for entry but nevertheless providing a clear path to improving standards (Box 2).

**Box 2:** Implementing MDAR at three tiers of stringency

MDAR implementation tiers	Example policy statement on journal/publisher policy sites
**Recommend:**	“XX endorse the Materials Design Analysis Reporting (MDAR) Framework for minimum reporting standards in the life sciences and encourage authors to consider all aspects of MDAR Framework (provide link to MDAR materials) relevant to study when submitting the study. Authors may submit the MDAR Checklist with their manuscripts.”
*Endorses MDAR Framework and recommends authors consider aspects of MDAR Framework relevant to study when submitting study.	
*MDAR Framework not mandatory and not enforced.	
*May encourage use of MDAR checklist	
**Limited mandate:**	“XX endorse the Materials Design Analysis Reporting (MDAR) Framework for minimum reporting standards in the life sciences and encourages authors to consider all aspects of MDAR Framework (provide link to MDAR materials) relevant to study when submitting the study. We require all studies using cell lines to adhere to the minimum reporting standards described in the MDAR framework. Authors may submit the MDAR Checklist with their manuscripts.”
*Endorses MDAR Framework and recommends authors consider aspects of MDAR Framework relevant to study when submitting study.	
*Additionally, commits to require limited aspects of MDAR Framework (see example with reference to cell lines)	
*May encourage use of MDAR checklist	
**Full mandate:**	“XX endorse the Materials Design Analysis Reporting (MDAR) Framework for minimum reporting standards in the life sciences. We require that reporting in life science papers adhere to all aspects of the minimum reporting standards set out in the MDAR Framework. Authors are encouraged/required to submit the MDAR Checklist with their manuscripts.”
*Endorses MDAR Framework.	
*Commits to require compliance on all aspects of MDAR Framework	
*May encourage or require use of MDAR checklist	

The MDAR Checklist is available as an optional resource, or tool, to help drive implementation of the Framework, but the Framework itself is purposely designed to be compatible with other modes of implementation including through structured queries in journal peer-review systems, integration within journal-specific guidelines or checklists, or automated or semiautomated manuscript processing systems.

Some journals already have policies that go beyond the minimum set of requirements and are aligned with the aspirational best practices described in the MDAR Framework. We expect that these journals will continue to implement their best practices while endorsing the Framework. Their experience will be central to inform future evolution of the minimum set of requirements in the MDAR Framework.

Some journals may wish to endorse the MDAR Framework but may be concerned about the burden on editorial resources. It is important to note that during the pilot in which editorial staff used the checklist to evaluate MDAR compliance the average time was 24 min (median 15 min). The assessment was largely carried out by internal journal staff and we did not test the checklist with volunteer reviewers or editors. However, as outlined in Box 2, the MDAR Framework can be recommended or required without the use of the checklist. One option could be that authors are asked to complete a checklist as part of their submission, with this being published as a supplement without monitoring compliance. This would raise awareness about reporting expectations, increase accountability for authors, and provide added value for readers but would require fewer editorial resources for implementation.

## What Comes Next?

The Center for Open Science (COS), which has been a partner in developing the MDAR Framework, will be responsible for stewardship, as a community resource, of the MDAR Framework and associated outputs including the Elaboration document and Checklist. COS will maintain a webpage to curate the checklist, Elaboration document, and any policy recommendations. These will be housed in a repository on the Metascience Collection on the Open Science Framework, which is developed and maintained by COS. The Metascience Collection contains data, materials, and research outputs relevant to the scientific community and enables resources such as the MDAR outputs to be maintained in version-controlled documents with persistent, unique identifiers. We hope to see widespread adoption of the MDAR Framework, and we ask journals and platforms to share details on this platform when they start to use the MDAR Framework.

Alignment around MDAR as a common standard for life-science papers will hopefully pave the way for the development of automated—and semiautomated—assessment tools that can determine manuscript adherence to reporting standards, potentially reducing barriers to wider uptake of the Framework (23). As the MDAR Framework is likely to evolve, we strongly encourage interested developers to focus not only on the minimum requirements but also on the aspirational best practices outlined in the Framework.

While journals are a key target audience, the MDAR Framework could be used by multiple stakeholders at different points in the research life cycle: by researchers during the design, conduct, analysis, and reporting of research studies; by institutions as a pedagogical tool to teach best practice in responsible conduct of research; and by funders and others involved in research assessment to help evaluate rigor and reporting transparency in grant applications, preprints, or research articles. With the growth in preprints in recent years, preprint servers are uniquely placed to collect (and post) an author-completed MDAR checklist, which could then travel with the manuscript when submitted to a journal. In all implementations it will be desirable to make the process of engagement with the MDAR Framework as straightforward for authors as possible.

Improved consistency in expectations for reporting across publishers has the potential to ease adoption of transparent reporting practices for authors, researchers, and journals and lead to improved quality of reporting in published papers. In addition, alignment on these expectations among funders could further help drive adoption and incorporation of best-practice recommendations early, into study design, data collection, and analysis. Ultimately the goal of transparent reporting is to improve research practice.

## Footnotes

*NASEM defines the term “reproducibility” as referring to computational reproducibility, so here we use the broader phrasing of “reproducibility and replicability.”
